# A Wearable and Deformable Graphene-Based Affinity Nanosensor for Monitoring of Cytokines in Biofluids

**DOI:** 10.3390/nano10081503

**Published:** 2020-07-31

**Authors:** Ziran Wang, Zhuang Hao, Shifeng Yu, Cong Huang, Yunlu Pan, Xuezeng Zhao

**Affiliations:** 1 Key Laboratory of Micro-Systems and Micro-Structures Manufacturing, Harbin Institute of Technology, Ministry of Education, Harbin 150080, China; 16B908024@stu.hit.edu.cn (Z.W.); hithuangcong@outlook.com (C.H.); yunlupan@hit.edu.cn (Y.P.); zhaoxz@hit.edu.cn (X.Z.); 2 School of Mechatronics Engineering, Harbin Institute of Technology, Harbin 150001, China; 3 School of Chemistry and Chemical Engineering, Harbin Institute of Technology, Harbin 150001, China; 4 Department of Mechanical Engineering, Columbia University, New York, NY 10027, USA; sy2892@columbia.edu

**Keywords:** wearable sensor, graphene field-effect transistor, cytokine, ultrathin sensor

## Abstract

A wearable and deformable graphene-based field-effect transistor biosensor is presented that uses aptamer-modified graphene as the conducting channel, which is capable of the sensitive, consistent and time-resolved detection of cytokines in human biofluids. Based on an ultrathin substrate, the biosensor offers a high level of mechanical durability and consistent sensing responses, while conforming to non-planar surfaces such as the human body and withstanding large deformations (e.g., bending and stretching). Moreover, a nonionic surfactant is employed to minimize the nonspecific adsorption of the biosensor, hence enabling cytokine detection (TNF-α and IFN-γ, significant inflammatory cytokines, are used as representatives) in artificial tears (used as a biofluid representative). The experimental results demonstrate that the biosensor very consistently and sensitively detects TNF-α and IFN-γ, with limits of detection down to 2.75 and 2.89 pM, respectively. The biosensor, which undergoes large deformations, can thus potentially provide a consistent and sensitive detection of cytokines in the human body.

## 1. Introduction

Human biofluids (such as saliva, tears and sweat) are attractive clinical diagnostic bio-media containing numerous cytokines (with a molecular weight lower than 70 kDa) [1,2,3]. They can be easily collected without skin-piercing. Abnormally elevated levels of cytokines in human biofluids are considered to be closely related to the attack of some severe diseases, such as coronavirus disease 2019 (COVID-19), and chronic diseases [4,5,6]. Hence, the capacity for the continuous detection of cytokine levels in human biofluids for high risk populations, daily, is of great significance for offering information on health conditions and thereby gaining valuable time, which can then be used to take effective preventative measures before the attack from such diseases [7,8]. Wearable sensors, which could be attached to the non-planar human body surface and complete on-site signal transduction, seem to be capable of performing such work.

Graphene, an attractive two-dimensional nanomaterial, is extremely sensitive to its surface charge distribution, and widely used as the transducer for sensors due to its outstanding electrical properties [9,10,11]. Especially, with the aid of aptamers, the graphene field-effect transistor (GFET) can enable the sensitive, rapid and label-free detection of cytokines [12,13,14]. To date, efforts have been made to use GFET biosensors in wearable applications due to the high mechanical flexibility of graphene [15,16,17]. Such sensors are fabricated on sheets of polymers, such as polydimethylsiloxane (PDMS), polyester (PET) and polyethylene naphthalate (PEN) [18,19,20]. However, these polymer sheets, whose overall thicknesses are 100 μm or more, would hardly be subject to large deformations and curvature (with radii ranging from 4 to 40 mm). As such, GFET biosensors with an extremely thin substrate that can sustain the large deformations involved in physiologically and biochemically relevant measurements on non-planar human body surfaces are still highly desirable.

**Figure 1:**
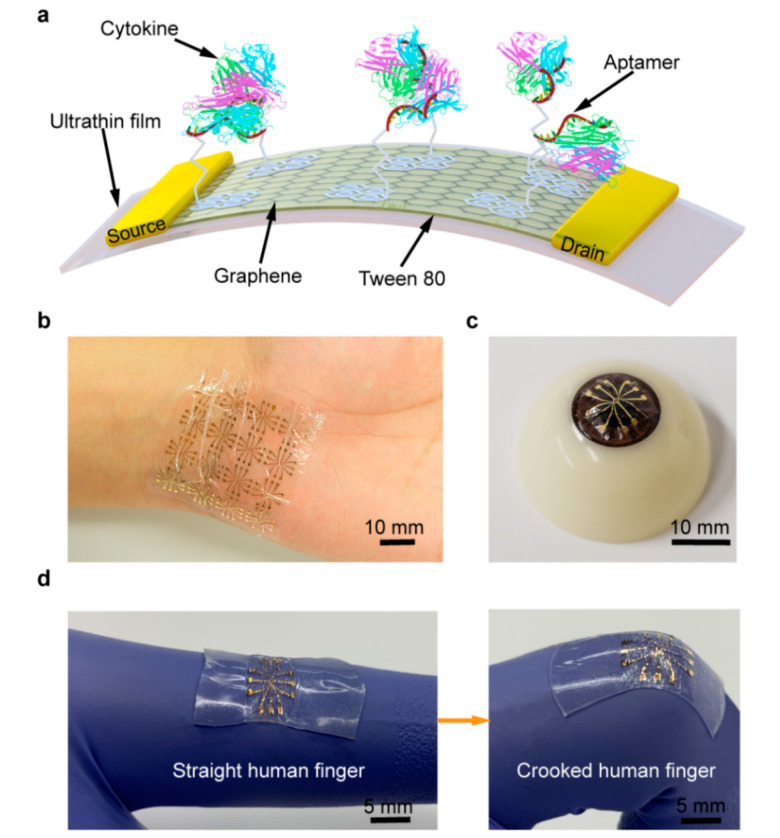
Ultra-flexible graphene field-effect transistor (GFET) biosensor. (**a**) Schematic of the GFET biosensor fabricated on an ultrathin film. Photograph of the flexible device conformably attached onto the (**b**) human wrist and (**c**) artificial eyeball. (**d**) Stretchable biosensor can be stretched with the activity of the human body.

In this paper, we present a wearable and deformable aptameric GFET biosensor that is designed to enable the sensitive, consistent and time-resolved monitoring of cytokines in human biofluids. The biosensor is fabricated on a biocompatible and ultrathin, polymer-supporting substrate (Figure 1a). Due to the employment of this substrate, whose thickness is only 2.5 μm, the biosensor, with good mechanical durability, is capable of conforming to non-planar surfaces such as the human skin or eyeball and withstanding large deformations, including bending and stretching, whilst maintaining consistent and sensitive responses. Moreover, Tween 80 is used to modify the graphene surface to effectively suppress nonspecific adsorption, thus enabling the biosensor to detect cytokines (TNF-α and IFN-γ, significant inflammatory cytokines, were used as representatives) in artificial tears (used as a biofluid representative). The experimental results indicate that our aptameric GFET biosensor can realize the highly sensitive detection of TNF-α and IFN-γ, with limits of detection down to 2.75 and 2.89 pM, respectively. Furthermore, the time-resolved monitoring of TNF-α in artificial tears under different tensile strains with consistent sensing responses is enabled. As a result, our biosensor can be potentially used in wearable applications for monitoring an individual’s health conditions and predicting the attack of chronic diseases.

## 2. Materials and Methods

### 2.1. Materials

The monolayer graphene sheet was ordered from Graphenea Inc. (Cambridge, MA, USA). 1-pyrenebutyric acid N-hydroxysuccinimide ester (PASE), ethanolamine, Tween 80, human interleukin-002 (IL-002), TNF-alpha and IFN-gamma were purchased from Sigma-Aldrich (St. Louis, MO, USA). Human growth hormone (GH) and human epidermal growth factor (EGF) were ordered from ACRO Biosystems (Newark, DE, USA). Ultrathin Mylar films (2.5 μm thickness of polyethylene terephthalate membranes) were ordered from Chemplex Industries (Palm City, FL, USA). Artificial tears were ordered from Alcon Laboratories. The aptamer specific to TNF-alpha (5′-NH_2_-TGG TGG ATG GCG CAG TCG GCG ACA A-3′) and IFN-gamma (5′-NH_2_-GGG GTT GTT TGT GTT GGG TGT TGT GT-3′) was synthesized and purified by Sangong Biotech (Shanghai, China).

### 2.2. Biosensor Design and Fabrication

The GFET biosensor was fabricated following our previous nanofabrication process [13,21,22]. Briefly, an ultrathin Mylar film (2.5 µm) was placed on a glass slide as the biosensor’s substrate. Subsequently, drain, source and gate electrodes (4/46 nm of Cr/Au) were patterned onto the film using a lithography process, including e-beam evaporation and lift-off. The biosensor was then exposed to the oxygen plasma to remove the residue on the surface. A monolayer graphene sheet was transferred onto electrodes as the conducting channel, using a polymethyl methacrylate (PMMA) carrier layer. After dissolving the PMMA layer with acetone, the graphene was biochemically functionalized to enable the biomarker detection. The fabricated biosensor was ultra-flexible and capable of conforming to the underlying surface, such as a human wrist or eyeball (Figure 1b,c). Finally, the biosensor was mounted onto a pre-stretched elastomer to obtain the necessary stretchability, which allowed the biosensor to be stretched from a 0% to 100% extension (Figure 1d).

### 2.3. Surface Functionalization

To achieve the biochemical functionalization, the biosensor was first immersed in 10 mM 1-pyrenebutanoic acid succinimidyl ester (PASE) solution for 5 h at room temperature. PASE was modified on the graphene surface through π-π stacking, which was used to link the aptamer. After incubating in the 1 µM aptamer solution for 12 h, the device was washed with phosphate buffer (PBS) to remove free aptamer. Ethanolamine was then used to quench the unreacted PASE on the graphene by soaking in 100 mM ethanolamine solution for 1 h. Finally, the biosensor was immersed in 0.05% Tween 80 solution to passivate the uncoated graphene area.

### 2.4. Liquid Handling

During the operation, a volume of 40 µL of analytes (TNF-α, IFN-γ and control proteins) at a given concentration was added to a polydimethylsiloxane (PDMS) open well, which was mounted on the graphene conducting channel to hold the analyte solution. In the experiments, 20 µL of artificial tears was added to a 1 mL centrifugal tube with 980 µL of 1 × PBS. Then, the mixture solution was stored at 4 °C before protein solution configurations.

## 3. Results and Discussion

### 3.1. Surface Characterization

**Figure 2:**
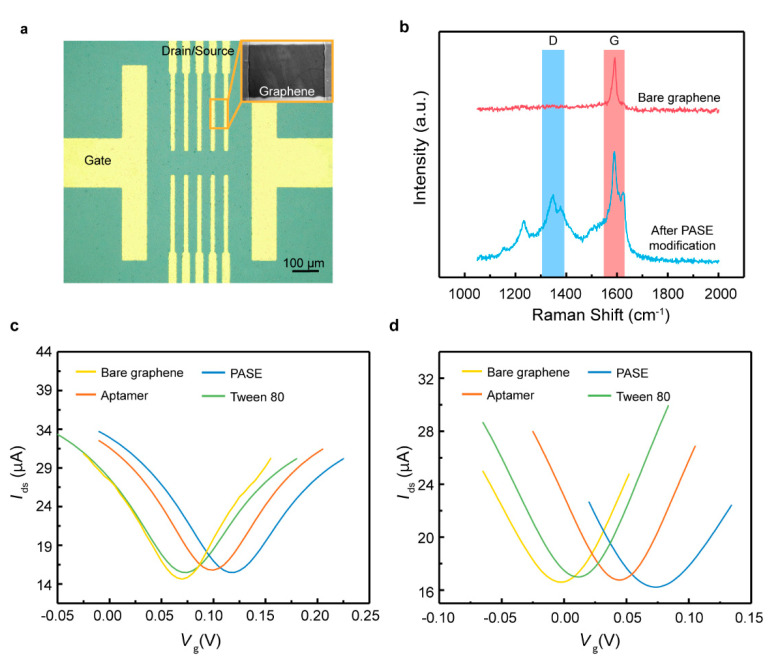
Functionalization of the GFET biosensor. (**a**) Microscope image of the flexible GFET biosensor with drain, source and gate electrodes. (**b**) Raman spectra of the graphene before and after PASE modification. Transfer characteristic curves of the graphene before and after PASE, (**c**) TNF-α aptamer, (**d**) IFN-γ aptamer, and Tween 80 treatment.

A microscope image of the GFET biosensor fabricated on the ultrathin film is shown in Figure 2a. The surface functionalization of the graphene with PASE was confirmed using Raman spectra (Figure 2b). The modification of the PASE split the G band, illustrating the coupling of graphene with the pyrene group on PASE. Furthermore, the Dirac point shift Δ*V*_Dirac_,where Δ*V*_Dirac_ = *V*_Dirac_ – *V*_Dirac,0_ with *V*_Dirac,0_ the Dirac point obtained in the solution without biomarker, before and after PASE, aptamer and Tween 80 functionalization was measured with the gate voltage *V*_g_ increasing from −0.2 to 0.4 V at a fixed drain-source voltage *V*_ds_ of 0.01 V (Figure 2c,d). Δ*V*_Dirac_ was observed to be monotonically increased after PASE functionalization, indicating that the p-type doping of the graphene had been induced. Upon the attachment of the aptamer specific to TNF-α and IFN-γ, Δ*V*_Dirac_ decreased by 0.04 and 0.057 V, respectively. Tween 80, a chemically stable nonionic surfactant, was used to block the uncoated graphene area to suppress the nonspecific adsorption in artificial tears due to its low binding affinity to the abundant non-target molecules present in the tears [23,24]. After the modification with Tween 80, the Dirac point *V*_Dirac_ shifted towards the direction of the negative gate voltage, illustrating that Tween 80 induced the n-type doping of the graphene. Thus, it was concluded that the biosensor was successfully functionalized using different aptamers.

### 3.2. Cytokine Detection in Artifical Tears

**Figure 3:**
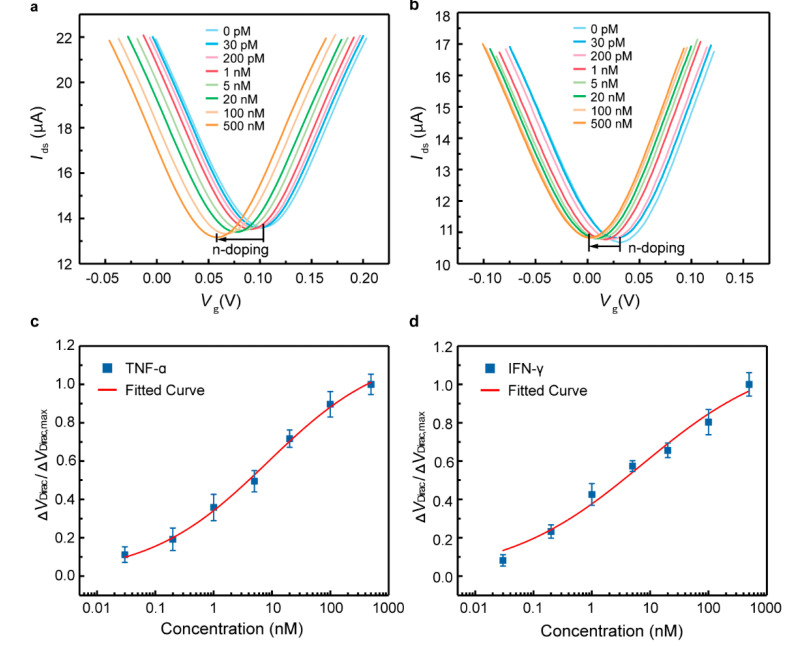
Cytokine detection in artificial tears. Transfer characteristic curves measured when the biosensor was exposed to (**a**) TNF-α and (**b**) IFN-γ solutions with various concentrations. The normalized Dirac point shift as a function of the (**c**) TNF-α and (**d**) IFN-γ concentration. The fitted curves were fitted using the Hill–Langmuir equation.

The capability of the biosensor for cytokine detection was assessed using TNF-α and IFN-γ (Figure 3)—inflammatory cytokines related to inflammation, COVID-19 and cancers [25,26]. As the TNF-α concentration increased from 0.03 to 500 nM (Figure 3a), *V*_Dirac_ decreased by 0.042 V, from 0.1 to 0.062 V. It could be observed that *V*_Dirac_ monotonically decreased with increasing TNF-α concentrations, suggesting the successful detection of TNF-α in artificial tears using the fabricated biosensor.

The equilibrium dissociation constant, defined *K*_D_, was investigated to study the binding affinity between the aptamer and TNF-α (Figure 3c). The normalized Δ*V*_Dirac_ was employed to address the effect of device-to-device variations, defined as Δ*V*_Dirac_/Δ*V*_Dirac,max_ (*V*_Dirac,max_ is the Dirac point corresponding to the maximum TNF-α concentration tested). Δ*V*_Dirac_/Δ*V*_Dirac,max_ was determined using the Hill–Langmuir equation [27]. Based on the fitted curve, the *K*_D_ was calculated to be 8.43 ± 1.67 nM. The limit of detection (LOD) was estimated based on the three-sigma rule, and the sigma was obtained from the standard deviation of the experimental and fitted data. The LOD was calculated to be 2.75 pM, which is many times lower than that for existing biosensors for TNF-α detection.

Subsequently, the biosensor was employed to test the detection of IFN-γ in artificial tears (Figure 3b,d). As the IFN-γ concentration increased from 0.03 to 500 nM, the maximum Δ*V*_Dirac_ was 0.029 V, decreasing from 0.029 to 0 V. The sensing signal was also consistently shifted in the direction of the negative gate voltage with the increasing IFN-γ concentrations. This was expected from the electrostatic mechanism [18]. After binding with the cytokines, the aptamer together with the negatively charged cytokines was brought closer to the graphene surface, which enabled the charge redistribution in the graphene, due to electrostatic induction [12,14]. Hence, a detectable change in the drain-source current was measured. The equilibrium dissociation constant *K*_D_ was estimated to be 7.36 ± 2.76 nM (Figure 3d), and the LOD was calculated to be 2.89 pM. Thus, the biosensor shows a consistent and sensitive response, able to detect cytokines in artificial tears with a lower LOD than most other existing methods (Table 1).

### 3.3. Specificity of the Biosensor in Artificial Tears

**Figure 4:**
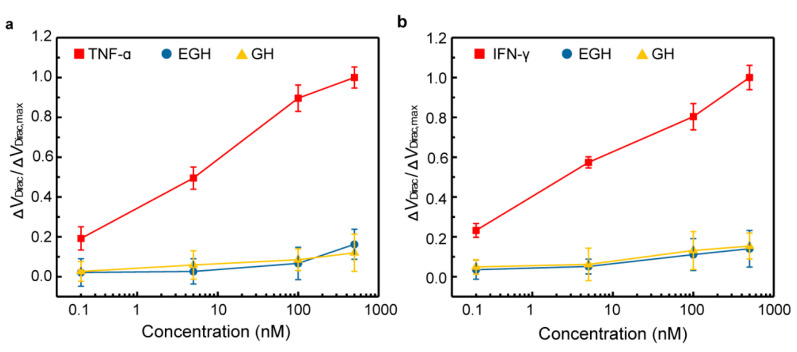
Specificity of the biosensor. The normalized Dirac point shift showing the sensing response of the biosensor to various concentrations (0.2, 5, 100 and 500 nM) of (**a**) TNF-α, (**b**) IFN-γ and control proteins (EGF and GH).

To investigate the specificity of the biosensor, EGF and GH, two related proteins, were chosen as control proteins. The biosensor, modified with the aptamer specific to TNF-α, was firstly exposed to control proteins in artificial tears (Figure 4a). The sensing signal for TNF-α significantly increased with the adding of TNF-α concentrations. The normalized Δ*V*_Dirac_/Δ*V*_Dirac,max_ for TNF-α at 500 nM was over six times larger than that for control proteins (14.3%) at the same concentration. Additionally, the dissociation constant *K*_D_ for the control proteins was investigated. *K*_D_ was estimated to be 1.43 × 10^10^ and 1.77 × 10^9^ nM for EGH and GH, respectively, which is much larger than the *K*_D_ for TNF-α (8.43 nM). Subsequently, the biosensor modified with the aptamer specific to IFN-γ was tested using these control proteins (Figure 4b). The Δ*V*_Dirac_/Δ*V*_Dirac,max_ for IFN-γ is also six times larger than those for the control proteins (15.6%) at the same concentrations. Furthermore, the *K*_D_ was calculated to be 1.55 × 10^6^ and 2.73 × 10^6^ nM for EGH and GH, respectively, which is tens of thousands of times larger than the *K*_D_ for IFN-γ (7.36 nM). Hence, the biosensor has a high level of specificity for the target cytokine in artificial tears.

### 3.4. Time-Resolved Experiments

**Figure 5:**
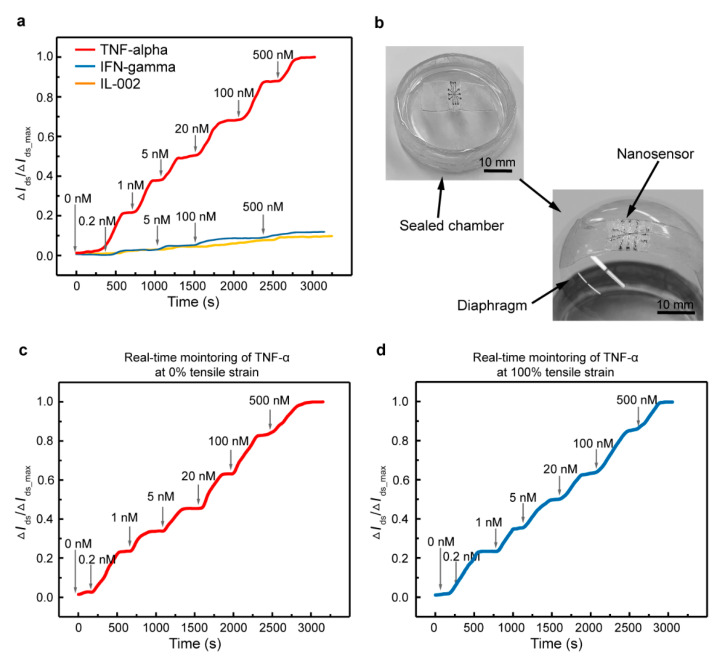
Time-resolved measurement of cytokines. (**a**) Real-time monitoring of TNF-α protein and control protein (IFN-γ and IL-002) in artificial tears. (**b**) Photograph showing the biosensor stretched by the inflated diaphragm chamber. Real-time monitoring of TNF-α using the biosensor at (**c**) 0% and (**d**) 120% tensile strain.

To verify the feasibility of the biosensor for wearable applications, time-resolved measurements were employed, using the biosensor modified with an aptamer specific to TNF-α as a representative. As shown in Figure 5a, the device was first exposed to different concentrations of TNF-α in artificial tears. The characterization started with the injection of the artificial tears in the PDMS well, then the artificial tears spiked with various TNF-α concentrations were injected after each binding equilibrium at corresponding concentrations. The changes in the drain-source current are denoted as Δ*I*_ds_, and the maximum change is denoted as Δ*I*_ds, max_. The sensing signal was observed to increase stepwise and significantly from 0 to 0.98 with increasing TNF-α concentrations from 0 to 500 nM. Moreover, it was found that equilibrium was reached after the binding of aptamer with TNF-α within 7 min at different concentrations. By contrast, the Δ*I*_ds/_Δ*I*_ds, max_—generated by related cytokines (IFN-γ and IL-002)—was less than 15% compared with that for TNF-α at the same concentration.

Additionally, we present a potential capability for wearable applications using the biosensor mounted on a homemade diaphragm chamber for cytokine detection (Figure 5b). The diaphragm is a stretchable membrane, which can be made to swell or shrink by pumping air into the chamber to mimic the human eyeball. The biosensor was first mounted on the planar diaphragm (0% tensile strain) to test cytokine detection (Figure 5c). Then, the diaphragm was inflated with compressed air until it achieved the shape of the human eyeball (which was accompanied by the biosensor, mounted on top). The diaphragm deflection induced a large-magnitude stretching of the biosensor (100% tensile strain), serving as an example mimicking the large deformation of the wearable device for cytokine detection on the human body (Figure 5d). The sensing response to TNF-α was sensitive and consistent with the test on a flat surface (Figure 5a), and the degradation of the signal was less than 10% at the same concentration for the biosensor under two tensile strains. Overall, the results demonstrate that the fabricated biosensor has promising prospects for being embedded with wearable devices for the monitoring of cytokines in body fluids.

## 4. Conclusions

We presented a wearable GFET biosensor for the time-resolved monitoring of cytokines in artificial tears. The modification of the graphene with the aptamer enabled the highly specific detection of the target cytokine. Tween 80, which occupies the uncoated area of the graphene, offers a capability of suppressing nonspecific adsorption; the biosensor hence affords a high level of sensitivity for cytokine detection in artificial tears, with a LOD down to 2.75 and 2.89 pM for TNF-α and IFN-γ, respectively. In addition, the time-resolved monitoring of TNF-α in artificial tears using the biosensor under different tensile strains has been demonstrated. The sensing response for the target cytokines is consistent with that found on the flat surface. These results demonstrate that this wearable GFET biosensor is a critical step toward the general application of sensors for the monitoring of disease biomarkers in the human body.

## Figures and Tables

**Table 1:** Comparison of the biosensor with existing methods for cytokine detection.

Analyte	Methods	Receptor	Samples	Limit of Detection
TNF-α	Graphene-based sensor	Aptamer	1 × PBS	26 pM [18]
TNF-α	Graphene-based sensor	Aptamer	1 × PBS	5 pM [13]
TNF-α	Electrochemical sensor	Aptamer	1 × PBS	32 pM [28]
TNF-α	Graphene-based sensor	Aptamer	Artificial tears	2.75 pM (this work)
IFN-γ	Graphene-based sensor	Aptamer	1 × PBS	83 pM [29]
IFN-γ	Electrochemical sensor	Aptamer	1 × PBS	37 pM [28]
IFN-γ	Graphene-based sensor	Aptamer	Artificial tears	2.89 pM (this work)

## References

[1] Cook E.B., Stahl J.L., Lowe L., Chen R., Morgan E., Wilson J., Varro R., Chan A., Graziano F.M., Barney N.P. (2001). Simultaneous measurement of six cytokines in a single sample of human tears using microparticle-based flow cytometry: Allergics vs. non-allergics. J. Immunol. Methods.

[2] Deak M.A., Cizza G., Eskandari F., Torvik S., Christie I.C., Sternberg E.M., Phillips T.M. (2006). Measurement of cytokines in sweat patches and plasma in healthy women: Validation in a controlled study. J. Immunol. Methods.

[3] Byrne M.L., O’Brien-Simpson N.M., Reynolds E.C., Walsh K.A., Laughton K., Waloszek J.M., Woods M.J., Trinder J., Allen N.B. (2013). Acute phase protein and cytokine levels in serum and saliva: A comparison of detectable levels and correlations in a depressed and healthy adolescent sample. Brain Behav. Immun..

[4] Bandodkar A.J., Jeang W.J., Ghaffari R., Rogers J.A. (2019). Wearable sensors for biochemical sweat analysis. Annu. Rev. Anal. Chem..

[5] Tai L.-C., Gao W., Chao M., Bariya M., Ngo Q.P., Shahpar Z., Nyein H.Y.Y., Park H., Sun J., Jung Y. (2018). Methylxanthine drug monitoring with wearable sweat sensors. Adv. Mater..

[6] Wei G., Hiroki O., Daisuke K., Kuniharu T., Ali J. (2019). Flexible electronics toward wearable sensing. Acc. Chem. Res..

[7] Liu G., Qi M., Hutchinson M.R., Yang G., Goldys E.M. (2016). Recent advances in cytokine detection by immunosensing. Biosens. Bioelectron..

[8] Adalsteinsson V., Parajuli O., Kepics S., Gupta A., Reeves W.B., Hahm J.I. (2008). Ultrasensitive detection of cytokines enabled by nanoscale ZnO arrays. Anal. Chem..

[9] Kim B.J., Jang H., Lee S.K., Hong B.H., Ahn J.H., Cho J.H. (2010). High-performance flexible graphene field effect transistors with ion gel gate dielectrics. Nano Lett..

[10] Geim A.K., Novoselov K.S. (2009). The rise of graphene. Nat. Mater..

[11] Choi D., Choi M.-Y., Choi W.M., Shin H.-J., Park H.-K., Seo J.-S., Park J., Yoon S.-M., Chae S.J., Lee Y.H. (2010). Fully rollable transparent nanogenerators based on graphene electrodes. Adv. Mater..

[12] Hao Z., Pan Y., Shao W., Lin Q., Zhao X. (2019). Graphene-based fully integrated portable nanosensing system for on-line detection of cytokine biomarkers in saliva. Biosens. Bioelectron..

[13] Wang Z., Hao Z., Yu S., Moraes C.G.D., Lin Q. (2019). An ultraflexible and stretchable aptameric graphene nanosensor for biomarker detection and monitoring. Adv. Funct. Mater..

[14] Hao Z., Pan Y., Huang C., Wang Z., Lin Q., Zhao X., Liu S. (2020). Modulating the linker immobilization density on aptameric graphene field effect transistors using an electric field. ACS Sens..

[15] Eda G., Fanchini G., Chhowalla M. (2008). Large-area ultrathin films of reduced graphene oxide as a transparent and flexible electronic material. Nat. Nanotechnol..

[16] Mannoor M.S., Tao H., Clayton J.D., Sengupta A., Kaplan D.L. (2012). Graphene-based wireless bacteria detection on tooth enamel. Nat. Commun..

[17] Yang Y., Yang X., Zou X., Wu S., Wan D., Cao A., Liao L., Yuan Q., Duan X. (2017). Ultrafine graphene nanomesh with large on/off ratio for high-performance flexible biosensors. Adv. Funct. Mater..

[18] Hao Z., Wang Z., Li Y., Zhu Y., Wang X., De Moraes C.G., Pan Y., Zhao X., Lin Q. (2018). Measurement of cytokine biomarkers using an aptamer-based affinity graphene nanosensor on a flexible substrate toward wearable applications. Nanoscale.

[19] Pradhan D., Niroui F., Leung K. (2010). High-performance, flexible enzymatic glucose biosensor based on ZnO nanowires supported on a gold-coated polyester substrate. ACS Appl. Mater. Interfaces.

[20] Gong S., Schwalb W., Wang Y., Chen Y., Tang Y., Si J., Shirinzadeh B., Cheng W. (2014). A wearable and highly sensitive pressure sensor with ultrathin gold nanowires. Nat. Commun..

[21] Hao Z., Zhu Y., Wang X., Rotti P.G., DiMarco C., Tyler S.R., Zhao X., Engelhardt J.F., Hone J., Lin Q. (2017). Real-time monitoring of insulin using a graphene field-effect transistor aptameric nanosensor. ACS Appl. Mater. Interfaces.

[22] Huang C., Hao Z., Qi T., Pan Y., Zhao X. (2020). An integrated flexible and reusable graphene field effect transistor nanosensor for monitoring glucose. J. Mater..

[23] Chang H.-K., Ishikawa F.N., Zhang R., Datar R., Cote R.J., Thompson M.E., Zhou C. (2011). Rapid, label-free, electrical whole blood bioassay based on nanobiosensor systems. ACS Nano.

[24] Huang Y., Dong X., Liu Y., Li L.-J., Chen P. (2011). Graphene-based biosensors for detection of bacteria and their metabolic activities. J. Mater. Chem..

[25] Fiedler U., Reiss Y., Scharpfenecker M., Grunow V., Koidl S., Thurston G., Gale N.W., Witzenrath M., Rosseau S., Suttorp N. (2006). Angiopoietin-2 sensitizes endothelial cells to TNF-α and has a crucial role in the induction of inflammation. Nat. Med..

[26] Wei X., Su J., Yang K., Wei J., Wan H., Cao X., Tan W., Wang H. (2020). Elevations of serum cancer biomarkers correlate with severity of COVID-19. J. Med. Virol..

[27] Ohno Y., Maehashi K., Matsumoto K. (2010). Label-free biosensors based on aptamer-modified graphene field-effect transistors. J. Am. Chem. Soc..

[28] Liu Y., Liu Y., Matharu Z., Rahimian A., Revzin A. (2015). Detecting multiple cell-secreted cytokines from the same aptamer-functionalized electrode. Biosens. Bioelectron..

[29] Farid S., Meshik X., Choi M., Mukherjee S., Lan Y., Parikh D., Poduri S., Baterdene U., Huang C.-E., Wang Y.Y. (2015). Detection of interferon gamma using graphene and aptamer based FET-like electrochemical biosensor. Biosens. Bioelectron..

